# Evaluating the Effect of Kaftrio on Perspectives of Health and Wellbeing in Individuals with Cystic Fibrosis

**DOI:** 10.3390/ijerph19106114

**Published:** 2022-05-17

**Authors:** Sean A. Aspinall, Kelly A. Mackintosh, Denise M. Hill, Bethany Cope, Melitta A. McNarry

**Affiliations:** Applied Sports, Technology, Exercise and Medicine (A-STEM) Research Centre, Faculty of Science and Engineering, Swansea University, Fabian Way, Swansea SA1 8EN, UK; 921125@swansea.ac.uk (S.A.A.); k.mackintosh@swansea.ac.uk (K.A.M.); denise.hill@swansea.ac.uk (D.M.H.); 982528@swansea.ac.uk (B.C.)

**Keywords:** chronic illness, lived experience, qualitative analysis, modulators, trikafta, semi-structured, self-identity, quality of life

## Abstract

Background: Modulator therapy represents a significant step forward in CF care and is expected to have a significant impact on the health and mortality of many individuals with CF. Studies have predominantly explored the physiological effects of modulator therapy on clinical outcomes, with little consideration of the individual lived experience of modulator therapy among adults with Cystic Fibrosis. Methods: To explore this, semi-structured interviews were conducted with 12 individuals currently taking Kaftrio, which were subsequently thematically analysed. Results: Three overarching themes were identified: (i) positive perception of Kaftrio, (ii) negative perception of Kaftrio, and (iii) the relationships with the clinical team. The experience of modulator therapy should be recognised as being unique to the individual, with perceptions of illness, self-identity, and outcomes strongly dictating the lived experience. Conclusions: There is a consensus that, while for many, the quality of life is evidently increased through the use of Kaftrio, this is not without its own challenges. This highlights the need for both individuals with CF and their clinical teams to learn to navigate this new disease landscape.

## 1. Introduction

Cystic Fibrosis (CF) is the most common and severe autosomal, recessive genetic disorder in the Caucasian population, currently affecting over 10,000 individuals in the UK [1]. Complications caused by a compromised function of the Cystic Fibrosis Transmembrane Regulator (*CFTR*) gene result in impaired chloride ion transport in the epithelial cells, affecting pancreatic, respiratory, gastrointestinal, reproductive, and skeletal function [2]. Despite the fact that CF is a multi-organ disease, respiratory failure represents the main cause of morbidity and mortality. Specifically, the absence or dysfunction of the CFTR protein results in abnormal mucus secretion, recurring infections, inflammation, airway obstruction and, ultimately, progressive decline in pulmonary function [3,4,5,6,7].

The majority of treatments available for CF treat the complications and symptoms associated with the disease, independent of the genetic defect. However, over the last decade, treatment has gradually been directed towards restoring CFTR protein function, thus targeting the underlying cause of the disease [8,9]. CFTR modulators are targeted therapies that increase the processing and delivery of CFTR to the cell surface (correctors) and increase the flow of ions through activated CFTR proteins at the cell surface (potentiators) [10]. The introduction of Ivacaftor for individuals with class III and residual function mutations (e.g., G551D), followed by Tezacaftor/Ivacaftor combinations in individuals homozygous for F508del, have evidenced significant improvements in sweat chloride, pulmonary function, body weight, and overall quality of life (QoL) [9,11,12]. As of June 2020, NHS England and the European Medicines Agency (EMA) approved the use of the highly effective triple modulator therapy Tezacaftor/Ivacaftor/Elaxacaftor in individuals over the age of 12-years who present with at least one F508del mutation [8].

It is widely purported that initiating triple modulator therapy (Kaftrio) will change the course of CF lung disease for many individuals. Indeed, triple modulator therapy has been shown to have beneficial effects not only in those with mild-to-moderate CF but also in those with more severe pulmonary status [13]. Whilst current research is unequivocal in its evidence for positive respiratory outcomes, we are yet to truly understand the effects that chronic triple therapy treatment has on patient outcomes related to mental health and quality of life. Di Mango et al. [14] reported that three months of treatment with Elexacaftor/Tezacaftor/Ivacaftor resulted in significant improvements across the majority of domains of the CFQ-R questionnaire, with the exception of emotional functioning, health perceptions, body image, and digestive symptoms. However, such objective data fails to explore the intricacies of the true patient experience, which remain unknown. Given the diverse nature of CF, it is imperative to understand patients’ perspectives of the impact of Kaftrio in order to inform and shape future clinical practice.

Whilst the introduction of modulator therapy brings hope for many, there remain many unanswered questions. Given the magnitude that such a therapy is expected to have on an individual’s day-to-day life, understanding the real-world experience is key. Prior to therapy, previous lived experience has described CF as unpredictable and challenging, with individuals striving for equality and desiring to experience the opportunities that their healthy peers take for granted [15]. As individuals begin to experience the physiological effects of the treatment, this study aims to explore the effect these changes have on an individual’s perception of reality and to what extent the modulator has changed their life beyond just the physical aspects.

## 2. Methods

Twelve individuals (10 Females; 28.1 ± 6.4 years) with CF were recruited via social media and provided informed consent to participate in the study. Eight individuals had received treatment for 6-months prior to the interview, two individuals for 10-months prior on compassionate grounds, and two individuals had been prescribed Kaftrio for over one year as part of phase 3 clinical trials. The study was approved by the Research Ethics Committee at Swansea University (Ref: MN_22-10-22).

To explore the individuals’ experiences and perceptions of Kaftrio (ELX/TEZ/IVA, Vertex Pharmaceutical), participants were asked to take part in a semi-structured interview with the first author via video conferencing software (Zoom, Zoom Video Communications Inc., San Jose, CA, USA) lasting up to 60 min. The flexible interview guide was devised in line with Evans et al. [16] to facilitate an in-depth discussion of the personal experiences of Kaftrio and was pilot tested to ensure that the questions were sensitive and appropriate. The interview involved the exploration of commonly reported benefits and side-effects of Kaftrio, with social challenges, disease management, anxiety, and identity explored in line with existing literature [17]. To maintain anonymity, individuals were given a pseudonym so as not to be identifiable within the manuscript. Whilst the interviews probed both positive and negative effects of Kaftrio, participants were prompted to expand on how these personal experiences shaped their perceptions of Kaftrio.

The interviews were recorded and subsequently transcribed verbatim. All of the data were analysed through qualitative content analysis by the first author [18]. The data were subject to line-by-line coding to identify appropriate and accurate themes. These codes identified features of the data that the first author considered pertinent to the research question. A member of the research team independently verified that these themes were reflective of the narrative, thus representing the data appropriately. The first author then identified quotes that were congruent with the overarching themes. These quotes were then grouped into subthemes which were aligned with the overarching themes and related to the overall story and research question. These extracts aimed to identify issues within the theme to provide a clear example of the individual’s point.

## 3. Results

The main themes identified were: (i) a positive perceptions of Kaftrio, (ii) a negative perceptions of Kaftrio, and (iii) the relationship with the clinical team, as shown in Table 1.

### 3.1. Positive Perceptions of Kaftrio

Of the study population, 10 individuals demonstrated positive perceptions towards Kaftrio, with many rereferring to an increase in quality of life as the main positive outcome. Health prior to Kaftrio therapy appeared to be a strong indicator of perception, with those of poorer health perceiving themselves to have more substantial increases in their quality of life.

#### 3.1.1. Improved Quality of Life

All individuals who identified an increased quality of life stated that they noticed the positive effect of Kaftrio on multiple aspects of day-to-day living within a few days of treatment commencement. Specifically, participants cited: (i) reduced coughing, (ii) reduced breathlessness, (iii) more energy, (iv) increased appetite, (v) improved sleep duration and quality, and (vi) the ability to complete daily tasks easier. Angie described:

“*…a week in I would say I didn’t cough… I’d get up in the morning and cough for hours… now I test my cough and be like, god can I actually still cough?”… Also, there’s a block that we walk now and I never in a million years thought I would ever be able to walk around that… now I walk around it and I’m like what? I’ve just walked that I can’t believe it.*”

Craig also noted the profound effect Kaftrio had on sleep quality:

“*…one of the major things is sleeping at night and not coughing. So, I would be tossing and turning and coughing… even when I went on antibiotics. My second night on Kaftrio… I just hadn’t coughed yet. Even to this day [over six months] it’s the same and it’s something I can’t get my head around.*”

One outcome that resonated with all participants, regardless of pulmonary function, was the obvious change in energy levels on a day-to-day basis. As an example, Ben, suggested:

“*I am fitter and healthier now than I’ve ever felt in my entire life. Like on Saturday I rode 50 miles on a bike. Could never comprehend that in my entire life. Like ever doing that.*”

#### 3.1.2. Pulmonary Function and the “Purge”

Prior to taking Kaftrio, many individuals expressed concern at watching their pulmonary function regularly decline. For those who had not yet experienced a significant decline in forced expiratory volume (FEV_1_), they believed it was ‘only a matter of time’ until their condition deteriorated significantly. Self-reported increases in pulmonary function varied substantially between individuals, ranging from 1 to 20%. For many, an increase in pulmonary function brought comfort that Kaftrio was making a substantial difference at a cellular level. Marin spoke of her recent changes in pulmonary function:

“*Pre-Symkevi I was 55%, Symkevi gave me 10%. Kaftrio pushed me to numbers I had had when I was 13 [years old]—the best was 83% and now it’s more around 78–80%... all those percentages mean a lot don’t they?*”

Prior to this increase, many individuals highlighted experiencing the infamous ‘purge’, whereby individuals expectorate large quantities of mucus from their lungs in the 24–72 h after they commenced Kaftrio treatment. Many individuals placed value on this as a sign that the medication was starting to work:

“*Had the purge, enjoyed it! It only lasted around 12–24 h, my lungs felt so much clearer than ever before, I thought wow this is fast acting but you don’t believe it somehow. My lung function increased straight away. I stopped coughing within a week.*”

However, the percentage increase in FEV_1_ was not the main positive health outcome expressed by participants. Many found value in the perception that Kaftrio would help preserve their lung function, thereby reducing anxiety associated with pulmonary function tests, as they now placed less significance on test outcomes given their increased quality of life. Individuals often professed that clinical perceptions of health and decisions on their care were determined based on pulmonary function alone, without taking the patient perceptions into consideration when evaluating health and quality of life. Indeed, an absence of acknowledgement concerning the effects that pulmonary function readings had on anxiety and an individual’s mood had led some individuals in the past to actively avoid clinic visits. Participants noted that a low FEV_1_ value during routine clinic visits when they felt otherwise well had been mentally challenging. Hence, critically, Kaftrio was suggested to provide some confidence that these decreases in FEV_1_ may be less frequent, with Cynthia discussing how recent infections and declines were hopefully not going to have too much of an impact now:

“*Over the last year I was suffering more with haemoptysis and I got MRSA (* *methicillin-resistant Staphylococcus aureus) and NTM (* *Nontuberculous mycobacteria) all in one year. I could see edging toward 30 [years old] that this is going to be the decline basically and I was hoping that it [Kaftrio] would sort of, not let it [lung function] go down too much.*”

#### 3.1.3. Reduced Rate of Exacerbations

A characteristic of CF is that many individuals experience frequent exacerbations that may require hospitalisation for intravenous (IV) antibiotic therapy. Depending on disease severity, individuals spoke of how they can experience IV therapy every four to 12 weeks. For Ben and Charlie, who had previously been under frequent antibiotic regimes, Kaftrio significantly reduced the need for hospital visits. Ben described:

“*They [the clinical care team] have tried to put me on IV’s at least four times this year because they said to me, you ‘normally’ have them every three months. I have now accepted to go on it, but [since Kaftrio] that will be almost a full year without them, which is unheard of for me.*”

Similarly, Charlie spoke of the decreased frequency of her hospital visits:

“*I was in hospital like four or five times per year… [on Kaftrio] about 14 months is the longest I have been without and that’s the longest it’s been for seven years.*”

Whilst individuals accept that their CF is not cured and that they may be prone to IV treatment in the future, the significant reduction in treatment burden brought the participants a sense of relief. Whilst the requirement for IV treatment echo’s life pre-Kaftrio, it is something Rosie is willing to live with if it remains as infrequent:

“*…the other part of me was like, if I only have IV’s once a year for the rest of my life, then that’s cool, I can totally accept that.*”

#### 3.1.4. A Sense of Normality: Independence, Opportunity and Hope

The accumulative perceived effects of improved quality of life and management of pulmonary function had substantial effects on the mental state of many individuals. Angie spoke of how she feels ever closer to living a ‘normal’ lifestyle—or a lifestyle closer to that of her peers:

“*It does gives us that chance, like I can be like everyone else….as a woman, I can start to think about having kids, the door is still open for me now… I have more chances to take risks. Whereas before, I would just not even consider attempting things… there is nothing there now to stop me, these things are in my hands.*”

Individuals commonly mentioned the increased *choice* they had. Cynthia believed that whilst choices were not specifically taken away from her, her CF played a substantial, subconscious role in the choices and decisions that she made:

“*With time extending [due to Kaftrio] it just means you are like, ‘oh well maybe I could get that retirement plan, maybe I should think about that’. I never got a lifetime ISA (Individual Savings Account) because I was never going to get to 50 [years] to use it. I thought I had another 15 years tops… So I am sort of allowing myself to think about these things, whereas otherwise I would put them in a box.*”

Individuals felt the opportunities they had in life had increased considerably. Videl, for example, explained that she is starting to make plans to participate in activities that her CF had previously placed restrictions on:

“*… so I have ridden dressage for years… and that has slowly decreased because of my health… I had lost sight of everything that was important to me because I was so poorly… I dreamt about it (competing) but now I am like, come on, you can do it… I feel like I am making up for lost time.*”

For most within the study, Kaftrio represented the catalyst for a new illness narrative that was characterised by a sense of hope. Hence, Kaftrio signified a ‘new start’, where CF did not play such a critical role in their life. Kaftrio also represented substantial advances in treatment in a short space of time, with the hope that this may just be the beginning of further successful treatment options until the point that diagnosis with CF is no longer a “*life sentence*”.

### 3.2. Negative Perceptions of Kaftrio

The experiences associated with the negative perceptions of Kaftrio centred around: (i) side effects, (ii) the removal of therapy, and (iii) a loss of identity. Whilst most individuals spoke of side effects, a truly negative experience was found in six individuals. It was noted that two individuals had had to cease Kaftrio therapy due to both physiological and psychological side effects.

#### 3.2.1. Side Effects: A Decrease in Quality of Life

Those who displayed predominately negative perceptions of Kaftrio had experienced serious side effects, wherein their quality of life had deteriorated below that of the pre-Kaftrio period. Katie noted that she had tried to persist with the treatment for 10-months despite side effects, which included debilitating migraines, ‘*brain fog*’, and sound sensitivity, but eventually chose to stop treatment as her quality of life had become so poor that getting out of bed was difficult:

“*I was elated. It felt like I didn’t have CF but the headaches started on day one—we know migraines now. I would have to go be in a dark quiet room. That helped me a little... I did not feel like I was on this planet. I forgot my date of birth and sound sensitivity was crazy, even talking became a struggle… I went on for 10 months, I felt ungrateful. I thought this was how my new life was supposed to be (before I decided to come off it). I have no regrets—it is easier to live with CF than on Kaftrio.*”

Rachel documented the significant effect Kaftrio had on her body image, perception of self, and confidence, to the point where, despite her improved pulmonary function, she chose to cease treatment in the hope that she could better manage her weight and mental health:

“*I felt every time I was going to clinic, it was a few more kilograms… then it reached the point where I was the heaviest I have ever been and was really not comfortable. My lungs got better but I couldn’t enjoy and reap the benefits because I was putting on all this weight. I was looking at myself and wanting to cry because I was so unhappy in how I looked.*”

#### 3.2.2. Removal: Returning to a Life Pre-Kaftrio

Unpleasant side effects were something that many individuals within the study were willing to live with, given the trade-off for long-term health benefits. However, others worried that the potential side effects would mean that they had to discontinue Kaftrio. Similarly, all participants were wary of the effect that Kaftrio had on their liver, with the worry that potential increases in liver enzymes would result in their team removing access to modulator therapy. Laura, who had CF-related liver disease, noted that Kaftrio had elicited major positive changes to her pulmonary health, but she was concerned that this might only be short-lived should her liver status change:

“*Back in 2020, around June, my liver disease became fatal and failed… after being on it [Kaftrio] for a couple of weeks… my liver function rose… for me it was very stressful… The liver thing is never going to get better, that’s always going to be there. I have got the fear that… because your liver function can just go up, I get scared in case they stop you… would I revert back to how I was and everything that has improved be ripped away from me?*”

Accordingly, as many participants had seen the positive changes that Kaftrio had on their day-to-day life, a number of participants lived in fear of Kaftrio subsequently being removed due to other health complications. For many, returning to a life pre-Kaftrio was now unimaginable, with Ben describing:

“*I think my main anxiety comes from the fact that I’ve now been given this opportunity or like dangled carrot of, look what your life could be like, and in the back of my mind is when is it going to go away. All the time.*”

These feelings were echoed by Marin:

“*…It’s all riding on it [Kaftrio] now… there’s no other alternatives… and if it stops working, where do you go, even in your head with that?*”

As a result, a sense of uncertainty around the future was something that the majority of individuals reported, regardless of their overall experience. These participants stated that the lack of available knowledge regarding the long-term efficacy of Kaftrio raised concerns that their health may start to deteriorate without warning. For older individuals, such as Katie and Marin, they referred to Kaftrio as their ‘last chance’, and Marin expressed a desire for additional understanding as to how their health may hypothetically look in the short-term future:

“*What happens if I go back to where I was [pre-Kaftrio]? This was the be all and end all, this was supposed to solve all my problems and if this doesn’t work then what? I have had to speak with a psychologist... It is [the worry] more the idea of what happens when this goes away—how long is that going to be there? Nobody knows.*”

Many described CF as a proverbial rollercoaster, with emotional highs and lows. The participants were accustomed to “looking over their shoulder” for negative health outcomes to present themselves, and Kaftrio was suggested to represent for some a scenario that was almost too good to be true and “*something that never happens to us [CF individuals]*”. As such, individuals consistently professed they were “*not allowing themselves to get carried away*”.

#### 3.2.3. Loss of Identity

For some, modulator therapy resulted in an “identity crisis” and a feeling of being overwhelmed. Four individuals noted an understanding as to the path in which their life was following pre-therapy; however, the prospect of an extended life left a lot of unanswered questions and thoughts regarding both short- and long-term goals. Individuals spoke about how their CF has always been ‘road mapped’ out, whereas, since Kaftrio, the road was unclear. Ben spoke about his struggles of having to alter his perception of self:

“*…this is how I summed it up. I completely lost my identity. Like, I didn’t know who I was or what I am doing or what is going on… I felt like my identity was my health and my job and now they are not the same.*”

Indeed, the concept of a loss of identity highlights that, for some, Kaftrio may represent a period of trauma in which individuals find it hard to manage or conceptualise their new health status. For two participants, the issues lay in having to disassociate with the person they were and the life they were used to when the future remained so uncertain.

Overall, the perceived negative impacts of Kaftrio found within this sample were mainly focused on the side effects, having the taste of a ‘normal’ life cruelly removed, and fear and uncertainty regarding a drug in its infancy, which left the participants unable to let themselves become “*too carried away*”.

### 3.3. Relationship with Clinical Teams and Psychological Support

As life with CF begins to change following Kaftrio, some participants called for a change in their clinical care, with participants expressing a desire for their clinical care teams to listen more to their views. For those participants who were more confident, Kaftrio initiated a desire to take charge of their health and make decisions they felt were in their best interest, with Rachel expressing her hope that her input could be taken seriously:

“*I want my input to mean something to my clinic team—at the moment they roll their eyes as if to say, oh here she goes again. I feel judged, I might not know the science, but they forget I am the one living with the condition daily. I am hoping that with Kaftrio I can have a firmer stance on things I do not agree with—I hope it gives me the chance to show them I actually am right.*”

However, Rachel spoke of how her previous experience with the clinical care team had prevented her from reaching out for the support she needed while taking Kaftrio:

“*[In the past] I called my psychology team nearly in tears, I was really struggling. All they told me was that the waiting list was long and that I would likely not be seen for at least eight weeks… if that was someone’s cry for help, there is no-one listening. With Kaftrio, I lost my identity straight away, I was overwhelmed and didn’t know where to turn—I didn’t even try to phone clinic as I knew no-one would answer.*”

Charlie also called for psychological support to be routinely available at clinic visits, alongside the dieticians, physiotherapists, and consultants:

“*The teams need to do more. This is a life changing event that we have just been told to be grateful for and get on with it—I don’t know how to get on with it.*”

For those struggling with weight and their perception of self, CF dietary care was quoted as being ‘*not the best*’, with individuals feeling as though the concept of nutrition/dieting and CF has a stigma attached to it, even in the face of Kaftrio. With the introduction of Kaftrio, some individuals within the current study were seeking methods to control their weight, though they were met with a lack of importance placed on weight and physique by their clinical team. Rachel spoke of her experience when she met her team to raise weight-related concerns:

“*I told them I was struggling with perception of self. I did not like how I looked, it was damaging my confidence. I have always struggled with weight and managed to lose some of it myself pre-Kaftrio. But when I gained weight on Kaftrio, the only thing they had to say to me was, ‘no don’t worry, … your lung function looks great’. That’s not what I needed to hear—I felt lost.*”

Other individuals acknowledged the great work their clinical care teams undertook for them, but there was a consensus that there needed to be a deeper empathetic understanding of the new psychological and physical challenges that come with the use of Kaftrio. Hence, there was a call from the participants for care teams to gain a further understanding of the existential concerns regarding the dramatic change of health status for many (but not all) in an extremely short time period.

### 3.4. A Message to the 10%

Finally, all of the participants explained their hope that, as with themselves, one day there would be a treatment available for the 10% whose genetic mutation does not support therapy with Kaftrio. Many referenced a phenomenon they described as similar to ‘survivor’s guilt’. They alluded to how difficult they would find it, looking in from the outside, whilst somebody with the ‘same’ condition was starting to make plans about their new life. Individuals currently taking Kaftrio alluded to the fact that they wanted to make the most out of Kaftrio and adopt further health-seeking behaviours in honour and respect of those that were not able to take Kaftrio. For those who had ceased treatment with Kaftrio, they felt guilty that they were choosing to discontinue something that another individual would wish to have. Importantly, Kaftrio was not simply seen as just ‘another treatment’ but as a gift that they had to cherish.

“*I’ve got so much guilt that I can’t think about it too much. I have this like, it’s not survivors’ guilt but something along those lines… They [the ineligible] are just watching it all unravel. They [Vertex] have to do something for them.*”

Accordingly, the participants receiving Kaftrio felt a sense of responsibility to those who were unable to receive the therapy. Given the positive changes that it had provided to many participants’ quality of life, they felt they owed it to those not taking Kaftrio to try and live life to the fullest. At the same time, all individuals expressed sorrow that not everyone would tolerate or could take Kaftrio. The individual messages aimed at the 10% were that of belief, hope, and a will to keep fighting, as all individuals believed it was only a matter of time before they had alternative treatment. For most, their personal adherence to Kaftrio was in honour to them as a way to ensure they never take this ‘gift’ for granted.

## 4. Discussion

By employing a qualitative approach, this study has offered a unique, in-depth insight into the lived experience of modulator therapy for CF individuals, highlighting the multi-faceted implications associated with the changing landscape of the disease and its treatment. The participant narratives revealed the positive impact that Kaftrio had on the individual’s disease state, with an improved overall quality of life and a significant reduction in ‘classical’ CF challenges. Moreover, the accumulative effect of these positive changes was reported as facilitating a sense of hope, normality, and independence, thereby allowing individuals to live a lifestyle which they considered to resemble that of their healthy peers. However, individuals also narrated negative experiences associated with the therapy, revealing significant inter-patient variability outside the physiological context [19]. The current narrative was ultimately dictated by an individual’s ability to tolerate the therapy, with individuals expressing feelings of fear and resignation. Some individuals expressed increased anxiety and distress in relation to uncertainty around the removal of therapy and, for some, dealing with a redefining of one’s identity. Regardless of perception, individuals mentioned their relationships with their clinical teams and called for additional counselling and psychological support to be offered to meet the new psychological needs of the individual, given the significant change in the landscape of the disease.

The implementation of Kaftrio represented a positive shift in the illness narrative of those who were able to tolerate the therapy. For example, for the first time, many individuals noted a sense of control and optimism for the future due to Kaftrio’s substantial effect on their quality of life. Indeed, many participants viewed Kaftrio as a second chance at life in which individuals had the opportunity to use previously negative experiences to reconstruct a positive self-transformation for the future they did not previously have [20]. Although the data on modulator therapy and life expectancy is not yet available, Kaftrio gave individuals hope and a representation of trust in an imagined future. Given the life-limiting nature of CF, individuals spoke of how they did not trust in their future enough (pre-Kaftrio) to believe they would reach milestones such as retirement and parenthood. The implementation of Kaftrio elicited a reality-based belief that a positive future did exist in which an individual could now plan for life events they previously perceived as impossible.

Drawing on experience, individuals recounted the limiting effect CF had on their life prior to modulator therapy, with many professing that they had lost sight of things that were important to them due to poor health status. The implementation of Kaftrio was the catalyst for a reduction in traditional CF challenges, which decreased the burden that CF had on individuals’ day-to-day lives. Gratitude was expressed for this sudden respite, with individuals speaking of how thankful they were to have their *lives back*. Longitudinal analysis has shown that gratitude is associated with a number of positive traits such as increases in self-esteem, satisfaction with life and fewer symptoms of depression [21,22], suggesting that as well as positive physical change, modulator therapy may also enhance an individual’s mental well-being. As in Davidai and Gilovich [23], CF forced individuals to focus on the obstacles and difficulties in life, given that they demanded immediate action. Kaftrio represented a metaphorical tailwind, in which individuals were given a reprieve [24] and a chance to focus on the things in life that bring them positive emotions. For those who felt they had been simply existing, Kaftrio represented an opportunity in which they could now truly live.

Although, for many, the experience of modulator therapy was a predominately positive one, for some, the sudden change in perspective had negative psychological effects. Whilst individuals expressed hope and gratitude, the narrative highlighted the difficulty of dealing with this rapid change in health, with individuals experiencing anxiety and fear, as seen in cancer survivors, in response to uncertainty, unanswered questions, and fear of relapse [25]. The current study identifies that modulator therapy may elicit feelings of anxiety associated with the overwhelming and uncertain future individuals now face in regards to themselves, their identity, and their future. As reported in those who receive a non-diagnosis of Huntington’s Disease, the prospect of a prolonged life can be one that is intimidating and stressful, as individuals perceive demands that they now must do something they were unprepared for, extraordinary and/or meaningful with their new, longer lives [26].

In the context of planning their new lives, some spoke of a loss of identity and a need to redefine their sense of self. As with other chronic conditions, one’s illness identity is dictated by the degree to which the disease has affected the way they see themselves and that the illness is integrated into one’s sense of self [27]. Whilst many professed a state of acceptance around their CF, for those who felt their illness dominated their identity, the shift in health, and thus identity, was described as “*almost post-traumatic*”. For those who experienced a change in identity, albeit one of positive health, there was a need for meaning reconstruction, which elicited emotions commonly associated with the grieving process and the loss of self [28]. It is worth noting, however, that whilst the initial loss of identity can be a negative experience, as individuals construct new meaning to their new lives, Tedeschi and Calhoun [29] argue that this process can breed positivity through the phenomenon of post-traumatic growth (PTG). Through continuous reappraisal of themselves, individuals who do experience PTG report feelings of becoming more resilient, confident, and independent whilst also developing a greater awareness of life’s fragilities [28]. Whilst many had developed coping strategies for their CF, they felt surprised and unprepared to deal with this emotion in response to a wholly positive event. Given that PTG can initiate feelings of stress and anxiety, the development of psychological programs promoting coping strategies should be considered in the management of life post-initiation of therapy, as it is likely that this initial transition is met with fear and uncertainty [30,31].

It is clear from this present study that the effect of modulator therapy had a strong impact on the relationship an individual had with their clinical team, with individuals stressing that their clinical teams failed to understand them as an individual or the effect their authoritarian decisions had on their physical and mental well-being. As such, many individuals felt that they did not have input in their own treatment decisions or did not feel confident in disagreeing for fear of judgement or guilt. This experience has been identified within the broader literature, with CF individuals often finding difficulty in communicating with their clinical teams due to how they discuss sensitive topics [32]. Whilst clinical teams understand CF as an *illness*; there is often a failure to understand how each individual conceptualises the disease [33]. Given that many individuals receiving Kaftrio will now have to contest with the phenomena of survivorship, it is key for clinical teams to consider the psychological effects that this can have. Common concerns within this narrative mirror that of cancer survivors, with issues around managing stress, fear of recurrence [or in this instance, treatment removal] and living with uncertainty [30]. As individuals begin to redefine their constructs of reality without the burden of CF, there is a need for clinical teams to understand the new existential concerns of living with CF to ensure the post-Kaftrio era is not defined by poor clinical relations. As such, this study has implications for service delivery, with a heavier focus needed on the team’s understanding of the complexities and new challenges facing those with CF [33], enabling more meaningful relationships with clinical teams. Whilst clinical care teams perceive the life-changing nature of Kaftrio to be one of positivity for the CF individual, it must be understood that simply increasing one’s quantity of life does not necessarily increase one’s psychological wellbeing and quality of life in the long term [33].

The present study also identified feelings of survivor’s guilt in both those who could and could not tolerate the therapy, a concept similarly reported in other life-limiting diseases [34,35]. The concept of survivor’s guilt has been defined into four specific areas: *altered identity*, *altered relationships*, *mental health* and *physical symptoms*, and *resolution* [26,36]. The narrative identified each of these depictions aside from resolution (e.g., the feelings of guilt disappearing). Whether survivor’s guilt underpins the psychological experience of modulators is yet to be established and cannot be concluded from this narrative. However, the study identified that individuals conceptualised survivor’s guilt according to their treatment experience. For those able to tolerate therapy, feelings centred around a sense of substitute guilt or unfairness in relation to those who are ineligible or could not tolerate the therapy. Indeed, individuals manifested feelings of guilt due to the fact that they could now begin a life less compromised by CF, whereas others are not able to have that luxury. Similarly, as described in Hutson et al. [36], this shift in identity may have further repercussions when considering identification with others and feelings of belonging within the CF community. Alternatively, where the treatment failed, feelings of guilt centred around the idea that they were *ungrateful* or had *wasted* a treatment from which others could have benefited. The given individuals were aware of the substantial cost associated with the therapy, and the guilt was further exacerbated with perceptions of their personal burden on the healthcare system being heightened. As the occurrence of survival guilt in the context of modulator therapy is yet to be understood, there is a need for further research to ascertain when it occurs and develop potential preventative methods to aid patients.

## 5. Limitations

The perceptions of modulator therapy from this qualitative study were based on the lived experiences of 12 individuals, which led to data saturation, lending credibility to the findings. Whilst individuals were interviewed from across the United Kingdom and, therefore, from multiple different clinics, the study did not explore patient perspectives from other countries in which modulator therapy is available. Furthermore, the study population included significantly more females than males; as such, we are unable to ascertain whether these perspectives are also affected by sex or gender. Given both factors, the generalisability of this information is potentially limited.

Although genetic mutation was discussed, this was not a variable which was explored in detail. As suggested in Varilek and Isaacson [33], further research is needed to ascertain whether there are differences in the experience of modulator therapy between genotypes.

## 6. Conclusions

For many individuals, Kaftrio represents ‘as close to a cure’ as CF individuals will have access to in their lifetime, with substantial changes in their quality of life, opportunities, and optimism for the future. However, this does not come without negatives, with individuals experiencing anxiety with regards to side effects, the efficacy of long-term treatment, and a fear of a return to life pre-Kaftrio. For the few who feel left behind, those whom it did not work for, or for the 10% who are not eligible, the message remains one of hope. An important overarching theme is that more needs to be provided by clinical teams to help manage the magnitude of effect that Kaftrio has both physically and mentally. Whilst individuals express gratitude toward Kaftrio, for many, that does not come with an absence of negative emotions.

## Figures and Tables

**Table 1:** Identified themes regarding individual’s perceptions of Kaftrio.

Perception of Kaftrio	
Dimension	Theme
Perceived Positive	Improved Quality of Life Pulmonary Function and the Purge Reduced Rate of Exacerbation A Sense of Normality
Perceived Negative	Side Effects Removal Loss of Identity
Relationships	Clinical Team Psychological Support

## Data Availability

The data presented in this study are available on request from the corresponding author. The data are not publicly available due to their sensitive nature.
